# Aprendizado da sexualidade em contexto de iniquidades e violências: relatos de aborto de adolescentes de uma favela no Rio de Janeiro, Brasil 

**DOI:** 10.1590/0102-311XPT129223

**Published:** 2023-11-10

**Authors:** Beatriz Galli, Claudia Bonan

**Affiliations:** 1 Ipas Brasil, Rio de Janeiro, Brasil.; 2 Instituto Nacional de Saúde da Mulher, da Criança e do Adolescente Fernandes Figueira, Fundação Oswaldo Cruz, Rio de Janeiro, Brasil.

O livro *Entre o Segredo e a Solidão: Aborto na Adolescência*^1^, de Wendell Ferrari, é uma contribuição valiosa para a discussão sobre sexualidade, gênero e aborto na adolescência. O autor apresenta os resultados de uma pesquisa acerca do aborto provocado entre adolescentes, em que são analisados relatos de dez meninas entre 12 e 17 anos que vivem em uma favela do Rio de Janeiro, Brasil. A obra traz reflexões importantes quanto à justiça reprodutiva, pois as histórias contadas pelas próprias jovens escancaram as assimetrias interseccionais de gênero, raça, classe, idade e território. Desse ângulo de análise, o tema é pouco conhecido, sobretudo em virtude da escassez de estudos que alcançam um olhar tão próximo dessa realidade.

A *Pesquisa Nacional de Aborto* de 2021 trouxe dados reveladores sobre a vida reprodutiva das jovens, mostrando que 52% das entrevistadas tinham 19 anos ou menos quando fizeram o primeiro aborto ^2^. Proporções mais altas foram detectadas entre aquelas com menor escolaridade, negras. indígenas e residentes em regiões mais pobres. A leitura de Wendell Ferrari nos permite espreitar a realidade por detrás desses números e vislumbrar a experiência dessas jovens.

O livro está estruturado em uma apresentação, quatro capítulos sobre os resultados e considerações finais. Na Apresentação, temos um panorama dos dados nacionais e internacionais sobre aborto e são discutidas as dificuldades de obtenção de informações confiáveis sobre o aborto provocado em contextos de ilegalidade. Apesar disso, estudos nacionais mostram a expressiva magnitude do processo, a correlação com a morbidade e a mortalidade materna e a maior ocorrência de desfechos negativos entre mulheres negras, menos escolarizadas e mais jovens ^3^.

A relevância do estudo de Ferrari é demonstrada pela própria observação de que o aborto provocado é pouco tratado na ampla literatura sobre gravidez adolescente, assim como questões específicas dos adolescentes são pouco esclarecidas nos estudos sobre o aborto no Brasil. Não é de somenos, experenciar uma gravidez, decidir interrompê-la e realizar um aborto em situação de clandestinidade e vulnerabilidade em um momento da vida de aprendizado da sexualidade.

No Capítulo 1, Ferrari faz uma análise do tema do aborto no Brasil, dos anos de 1970 até a década mais recente, colocando em análise os diferentes espaços e atores participantes do debate. Autoridades governamentais, organizações partidárias, legisladores, operadores do direito, setores médicos, pesquisadores, autoridades religiosas, mídia e movimentos sociais, especialmente feministas e movimentos de saúde são alguns dos atores envolvidos nas contendas quanto ao tema. Observa o autor que no plano da produção de conhecimentos e desenvolvimento de políticas de assistência às situações de aborto veem-se alguns avanços, contudo obstáculos importantes persistem e podem ser exemplificados pelas polêmicas sobre o direito de interromper a gestação no contexto da epidemia de Zika vírus. Além disso, o horizonte de retrocessos está sempre à espreita.

Ferrari observa que os debates sobre reprodução e adolescência tendem a se centrar na gravidez como fato dado, não considerando a questão da gestação indesejada, os processos de decisão sobre mantê-la ou não, as estratégias para viabilizar a decisão de interromper, as dificuldades em virtude da menoridade civil, as relações desiguais de gênero, a falta de autonomia financeira, a falta de informações e conhecimentos sobre o procedimento abortivo, entre outras coisas.

No Capítulo 2, o caminho metodológico é detalhado. É muito interessante o relato sobre a construção do campo, as estratégias de aproximação com as adolescentes e o estabelecimento do vínculo de confiança, condição sine qua non para que lhe contassem experiências de iniciação sexual, gravidez e aborto. Psicólogo de formação, trabalhando no ambulatório de uma ONG atuante na favela, Ferrari inicialmente se propôs a separar o psicólogo do pesquisador, mas teve a sensibilidade de se render à dinâmica relacional que as próprias adolescentes impuseram, em que a separação rígida - e artificial - entre os dois papéis não parecia ser relevante. O método e técnicas escolhidos teve como preocupação central permitir que as histórias de aborto fluíssem a partir da perspectiva das jovens entrevistadas, e o modo de conduzi-los foi magistral. Também nesse aspecto, a obra de Ferrari é altamente recomendada a pesquisadora(e)s do aborto e, de modo geral, dos temas que envolvem sexualidade, reprodução e gênero.

As histórias contadas pelas adolescentes são apresentadas e discutidas nos Capítulos 3 e 4. A opção do autor de apresentar partes abrangentes de seu acervo de entrevistas no Capítulo 3 foi muito oportuna. Os leitores são expostos não apenas às análises, mas às falas das adolescentes, surtindo um efeito de maior proximidade com elas, conforme pretendido por Ferrari. Ao lê-las, temos um contato mais vívido com suas experiências e somos convidadas a participar ativamente da discussão do material. Os relatos são muito tocantes, ninguém sai incólume. Passam por vários temas como o processo de iniciação sexual, masturbação, dinâmicas das relações com parceiros afetivo-sexuais, usos de contraceptivos, experiência de engravidamento, decisão pelo aborto, itinerários para realizá-lo. Sentimentos múltiplos, ambíguos e conflitantes estão envolvidos nesses itinerários, tais quais culpa e alívio, solidão e solidariedade, estigmatização e afirmação da própria autonomia.

No Capítulo 4, Ferrari discute como, a partir da gravidez indesejada, as adolescentes buscam estratégias para acessar o procedimento do aborto, com os poucos recursos que dispõem, mesmo correndo diversos riscos. O abandono pelos parceiros, antes ou depois do aborto, é um componente traumático da experiência. Muitas vezes são parceiros com idade significativamente maior, pertencentes a outra classe social ou casados. Com eles, as jovens mantêm vínculos afetivo-sexuais assimétricos, com autonomia limitada para decidir quando e como ter - ou não - relações, negociar o uso da camisinha e, em face de uma gestação, levá-la adiante ou interrompê-la.

Outro componente da experiência das adolescentes é o sentimento de culpa que se acentua em contextos sociais e familiares em que o tema do aborto é silenciado ou associado ao pecado pela visão religiosa. O estigma social reforça o segredo e a solidão do aborto na adolescência, apontando a extrema fragilidade e sofrimento das jovens em suas experiências individuais e trajetórias abortivas. São relatos de dor, desamparo e solidão, antes e depois do aborto. O aborto ilegal é realizado mesmo com todos os riscos envolvidos, seja da clínica da favela, clínicas de outros bairros ou com o uso de medicamento sem origem conhecida comprado na própria favela. Nesse último caso, o autor sublinha novamente o contexto de opressão de gênero ao discutir relatos das adolescentes, uma vez que elas não podem comprar diretamente o medicamento para abortar na favela, pois nesse caso a compra é restrita aos homens, ou seja, uma forma de controle da sexualidade feminina.

As meninas sozinhas ou apoiadas por amigas devem arcar com os cuidados de saúde necessários no resguardo, por exemplo repouso, medicamentos para dor e sangramento. As redes de amizade e a Internet são os meios prioritários pelos quais elas acessam a informação para cuidar da própria saúde sexual e reprodutiva. O medo de serem julgadas, maltratadas, denunciadas ou presas afasta as mulheres dos serviços de saúde para cuidados pré e pós abortamento, sobretudo se forem mulheres negras e periféricas ^4^.

Nesse emaranhado de vivências, é possível perceber também resiliência e resistência; o aborto pode ser de certa forma libertário para as adolescentes. Em geral, o sentimento é de alívio com a interrupção da gestação, seja para perseguirem seus projetos de vida, seja para se livrar de um vínculo marcado por opressões e violências. Nenhuma adolescente relatou arrependimento. Conforme Ferrari sugere, nas Considerações Finais, o direito ao aborto é central para o processo de autonomia das mulheres.

Para jovens negras e periféricas, o aprendizado da sexualidade se dá em contextos de iniquidades e violências de gênero, raça e classe. O cenário desnudado pela pesquisa de Ferrari é de injustiça estrutural, com suas expressões nas vivências afetivas, sexuais e reprodutivas das adolescentes. O livro é leitura necessária e indispensável para pesquisadores, ativistas, profissionais de saúde e todas as pessoas que têm interesse em desvendar o contexto social caracterizado pela falta de acesso à saúde, desigualdade de gênero, racismo estrutural, clandestinidade e ilegalidade do aborto que atinge com maior impacto as adolescentes negras moradoras das periferias urbanas.
